# Ginger essential oil and citral ameliorates atherosclerosis in ApoE^−/−^ mice by modulating trimethylamine-N-oxide and gut microbiota

**DOI:** 10.1038/s41538-023-00196-0

**Published:** 2023-05-20

**Authors:** Suraphan Panyod, Wei-Kai Wu, Sin-Yi Peng, Yea-Jing Tseng, Ya-Chi Hsieh, Rou-An Chen, Huai-Syuan Huang, Yi-Hsun Chen, Hsiao-Li Chuang, Cheng-Chih Hsu, Ting-Chin David Shen, Kai-Chien Yang, Chi-Tang Ho, Ming-Shiang Wu, Lee-Yan Sheen

**Affiliations:** 1 Institute of Food Science and Technology, National Taiwan University, Taipei, Taiwan, ROC; 2 Center for Food and Biomolecules, National Taiwan University, Taipei, Taiwan, ROC; 3 Department of Medical Research, National Taiwan University Hospital, Taipei, Taiwan, ROC; 4 Department of Internal Medicine, College of Medicine, National Taiwan University, Taipei, Taiwan, ROC; 5 National Laboratory Animal Center, National Applied Research Laboratories, Taipei, Taiwan, ROC; 6 Department of Chemistry, National Taiwan University, Taipei, Taiwan, ROC; 7 Division of Gastroenterology, Perelman School of Medicine, University of Pennsylvania, Pennsylvania, PA USA; 8 Department and Graduate Institute of Pharmacology, College of Medicine, National Taiwan University, Taipei, Taiwan, ROC; 9 Department of Food Science, Rutgers University, New Brunswick, NJ USA; 10 Department of Internal Medicine, National Taiwan University Hospital, Taipei, Taiwan, ROC; 11 National Center for Food Safety Education and Research, National Taiwan University, Taipei, Taiwan, ROC

**Keywords:** Microbiota, Atherosclerosis, Nutrition

## Abstract

Recently, the role of the gut microbiota in diseases, including cardiovascular disease (CVD), has gained considerable research attention. Trimethylamine-N-oxide (TMAO), which is formed during ʟ-carnitine metabolism, promotes the formation of atherosclerotic plaques, causing thrombosis. Here, we elucidated the anti-atherosclerotic effect and mechanism of ginger (*Zingiber officinale* Roscoe) essential oil (GEO) and its bioactive compound citral in Gubra Amylin NASH (GAN) diet with ʟ-carnitine-induced atherosclerosis female ApoE^−/−^ mice. Treatment with GEO at both low and high doses and citral inhibited the formation of aortic atherosclerotic lesions, improved plasma lipid profile, reduced blood sugar, improved insulin resistance, decreased plasma TMAO levels, and inhibited plasma inflammatory cytokines, especially interleukin-1β. Additionally, GEO and citral treatment modulated gut microbiota diversity and composition by increasing the abundance of beneficial microbes and decreasing the abundance of CVD-related microbes. Overall, these results showed that GEO and citral may serve as potential dietary supplements for CVD prevention by improving gut microbiota dysbiosis.

## Introduction

Cardiovascular disease (CVD) is a severe health problem and one of the leading causes of death globally^1^. The primary causes of CVD includes unhealthy diet and insufficient physical activity^2^. The gut microbiota and its metabolites are strongly associated with dietary intake and have recently been identified as an emerging risk factor for CVD^3^. High animal-protein consumption is a primary inducer of CVD risk factors and is associated with CVD^3,4^. Several food components from red meat, poultry, and dairy products, such as phosphatidylcholine, choline, and ʟ-carnitine, can be metabolized via meta-organismal metabolism involving specific gut microbiome and host to form γ-butyrobetaine (γBB), trimethylamine (TMA), and trimethylamine-N-oxide (TMAO)^5^. Additionally, TMA can be subsequently oxidized to TMAO by the host hepatic flavin monooxygenase^6^, and high blood TMAO levels are associated with cardiovascular events and mortality^7^. The atherogenic effects of TMAO include increased foam cell formation, reduced reverse cholesterol transport, enhanced platelet aggregation, and promotion of kidney fibrosis^6,8,9^; moreover, TMAO also causes vascular inflammation^10^.

Obesity is strongly linked to CVD^11^. In animals, mice fed high-fat diet (HFD) showed elevated levels of circulating TMAO, which was associated with cardiac dysfunction^12^. Moreover, an HFD animal model with gut microbiota imbalance is essential for studying the gut-liver axis^13^. Recently, an emerging Western diet called Gubra Amylin NASH diet (GAN diet) has been developed to induce several high cholesterol-related diseases, including obesity and CVD. GAN diet is composed of 40 kcal% fat (primarily from palm oil), 20 kcal% fructose, and 2% cholesterol^14^. GAN diet-fed mouse model exhibits the advantage of translatability to humans in terms of liver biopsy phenotype^15^. Thus, combining GAN diet with ʟ-carnitine may potentially elevate blood TMAO levels and allow the investigation of atherosclerosis-related mechanisms. Atherosclerosis-prone apolipoprotein E-deficient (ApoE^−/−^) mouse model is a valuable tool for studying the development of atherosclerotic plaques. ApoE^−/−^ mice are deficient in the ability to clear lipoproteins, resulting in the accumulation of cholesterol ester-enriched particle in blood, which can promote atherosclerosis^16^.

Preventive medicine is the practice of promoting preventive health care to avert disease development. Several foods and herbs can alleviate the progression of CVD and atherosclerosis^3,17^. Ginger is a herbal dietary component with potential benefits against CVD, including reducing total blood cholesterol and pro-inflammatory cytokine levels and improving insulin resistance^18^. Ginger essential oil (GEO), the volatile oil obtained from fresh ginger, can reduce plasmas levels of total cholesterol and total triglycerides and insulin in HFD-induced obese mice^19^. Citral, a primary components of GEO, has been shown to exert anti-obesity effect^19^. Moreover, GEO ameliorated liver inflammation in GAN diet-induced non-alcoholic steatohepatitis (NASH) mouse model via NLR family pyrin domain-containing 3 inflammasome (NLRP3)-mediated modulation of gut microbiota-lipopolysaccharide (LPS)/toll-like receptor 4 (TLR4) signaling pathways, and by increasing the abundance of beneficial microbes^20^. However, studies on the anti-atherosclerosis effect of GEO and citral are limited, and it is on this basis that this study aims to examine the effect of GEO and citral in GAN diet/ʟ-carnitine-induced atherosclerosis ApoE^−/−^ mice.

## Results

### GEO and citral protects against atherosclerosis and improve plasma lipidemic biomarkers

**Fig. 1:**
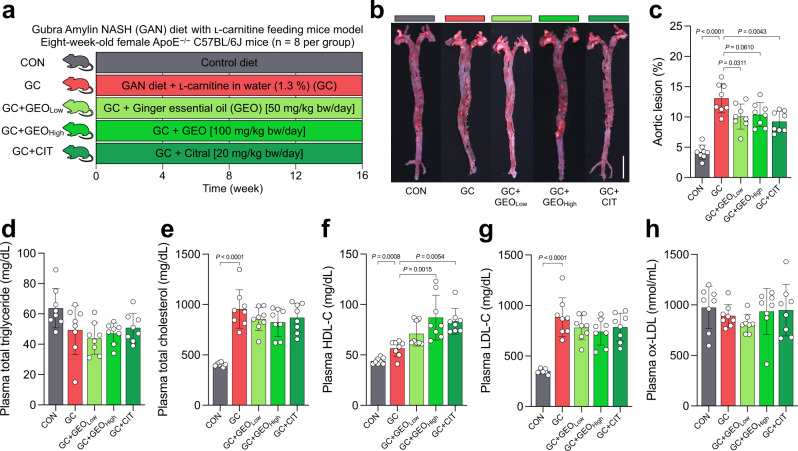
GEO and citral reduced aortic lesions and affected plasma lipid biomarkers in the GC-induced atherosclerosis female ApoE^−/−^ mice (n = 8 per group). **a** Experimental design; **b** representative image of oil red O stained aorta, scale bar is 0.5 cm; **c** percentage of aortic lesions; **d** plasma levels of total triglyceride; **e** total cholesterol; **f** high-density lipoprotein cholesterol (HDL-C); **g** low-density lipoprotein cholesterol (LDL-C); **h** oxidized low-density lipoprotein (ox-LDL). Dot plots are expressed as the mean ± standard deviation (SD). Statistical analyses were performed using an unpaired two-tailed Student’s *t*-test, CON vs. GC groups; one-way analysis of variance (ANOVA) with Tukey’s range test for comparing GC, GC + GEO_Low_, GC + GEO_High_, and GC + citral (CIT). CON: control diet group, GC: Gubra Amylin NASH diet [GAN diet] + ʟ-carnitine in drinking water [1.3%] group, GC + GEO_Low_: GC + Ginger essential oil (GEO) [50 mg/kg bw/day] group, GC + GEO_High_: GC + GEO [100 mg/kg bw/day] group, GC + CIT: GC + Citral [20 mg/kg bw/day] group.

To examine the protective effect of GEO and citral against CVD and atherosclerosis, ApoE^−/−^ mice were fed GAN diet + water supplemented with 1.3% ʟ-carnitine (GC) to induce atherosclerosis, and treated with GEO (Low: 50 mg/kg bw and High: 100 mg/kg bw) or citral (20 mg/kg bw) daily (Fig. 1a). After 16 weeks, the mice were sacrificed and aortic lesions were visualized using oil red O staining (Fig. 1b). Aortic lesion formation significantly increased (*p* < 0.0001) by 212% (13.1 ± 2.4%) in the GC group compared with control (CON) group (4.2 ± 1.2%), indicating successful induction of atherosclerosis using GAN diet and ʟ-carnitine (Fig. 1c). However, treatment with low-dose GEO, high dose GEO, and citral significantly reduced the occurrence of aortic plaques by 23% (*p* = 0.0311), 20% (*p* = 0.0610), and 29% (*p* = 0.0043), respectively. Additionally, the plasma levels of lipidemic biomarkers, including total triglyceride, total cholesterol, high-density lipoprotein cholesterol (HDL-C), low-density lipoprotein cholesterol (LDL-C), and oxidized low-density lipoprotein (ox-LDL), were examined (Fig. 1d–h). Compared with the CON group, plasma cholesterol (*p* < 0.0001), HDL-C (*p* = 0.0008), and LDL-C (*p* < 0.0001) levels were significantly elevated in the GC group, indicating that this intervention affected the circulation of lipids. However, there was no significant difference in plasma total triglyceride and ox-LDL levels between the CON and GC groups. Treatment with GEO and citral caused a decreasing trend in plasma cholesterol and LDL-C levels in ApoE^−/−^ mice compared with the GC group; moreover, high-dose GEO and citral increased plasma HDL-C levels (*p* = 0.0015 and *p* = 0.0054, respectively). However, there were no significant differences in the plasma levels of several biomarkers between low and high GEO groups, which may be because the effect of GEO on plasma biochemical indices peaked at 50 mg/kg bw/day and higher doses did not induce any significant change. Overall, these results showed the GEO and citral prevented atherosclerotic lesion formation and improved plasma lipid profile.

### GEO and citral improves glucose and insulin homeostasis, hepatic function, and plasma pro-inflammatory cytokine levels

**Fig. 2:**
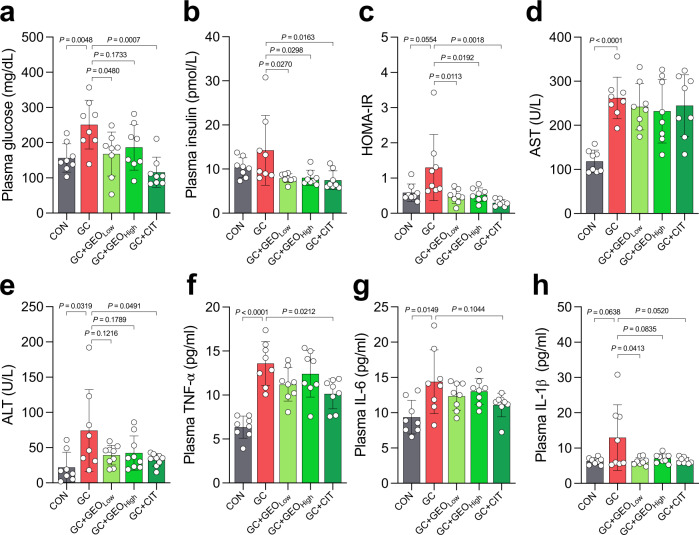
GEO and citral improved glucose homeostasis and hepatic function and alleviated systemic inflammation in GC-induced atherosclerosis female ApoE^−/−^ mice (*n* = 8 per group). Plasma levels of (**a**) glucose and (**b**) insulin; (**c**) homeostasis model assessment for insulin resistance (HOMA-IR); **d** aspartate aminotransferase (AST); **e** alanine aminotransferase (ALT); **f** tumor necrosis factor-α (TNF-α); **g** interleukin 6 (IL-6); and **h** interleukin-1β (IL-1β). Dot plots are expressed as the mean ± SD. Statistical analyses were performed using an unpaired two-tailed Student’s *t*-test, CON vs. GC groups; one-way ANOVA with Tukey’s range test for comparing GC, GC + GEO_Low_, GC + GEO_High_, and GC + CIT. CON: control diet group, GC: Gubra Amylin NASH diet [GAN diet] + ʟ-carnitine in drinking water [1.3%] group, GC + GEO_Low_: GC + Ginger essential oil (GEO) [50 mg/kg bw/day] group, GC + GEO_High_: GC + GEO [100 mg/kg bw/day] group, GC + CIT: GC + Citral [20 mg/kg bw/day] group.

Furthermore, the effect of GC, GEO, and citral on glucose and insulin homeostasis, hepatic function, and plasma pro-inflammatory cytokine levels were examined. Homeostatic model assessment-insulin resistance (HOMA-IR) index was calculated to determine the effect of the treatments on insulin resistance. HFD has been shown to negatively affect glucose and insulin metabolism^21^. Plasma glucose (*p* = 0.0048) and insulin (*p* = 0.2019) levels and HOMA-IR index (*p* = 0.0554) were substantially higher in the GC group compared with the CON group (Fig. 2a–c). However, low-dose and high-dose GEO, and citral improved plasma glucose (*p* = 0.0480, *p* = 0.1733, and *p* = 0.0007, respectively) and insulin (*p* = 0.0270, *p* = 0.0298, and *p* = 0.0163, respectively) levels and HOMA-IR values (*p* = 0.0113, *p* = 0.0192, and *p* = 0.0018, respectively). Plasma levels of hepatic parameters, including aspartate aminotransferase (AST; *p* < 0.0001) and alanine aminotransferase (ALT; *p* = 0.0319), increased by 2.2- and 3.4-fold, respectively, in the GC group compared with the CON group (Fig. 2d, e). However, treatment with low-dose and high-dose GEO and citral substantially ameliorated the ALT levels (*p* = 0.1216, *p* = 0.1789, and *p* = 0.0491, respectively), but did not affect AST levels. Compared with the CON group, there was a significant increase in plasma levels of pro-inflammatory cytokines in the GC group, including tumor necrosis factor-α (TNF-α, *p* < 0.0001), interleukin 6 (IL-6, *p* = 0.0149), and interleukin-1β (IL-1β, *p* = 0.0638). However, treatment with low and high doses of GEO tended to lower plasma TNF-α and IL-6 levels and significantly reduce IL-1β levels (*p* = 0.0413 and *p* = 0.0835, respectively). Moreover, treatment with citral significantly reduced plasma TNF-α (*p* = 0.0212), IL-6 (*p* = 0.1014), and IL-1β (*p* = 0.0520) levels. Overall, these findings showed that both GEO and citral have the potential to alleviate systemic inflammation, hepatic damage, and insulin resistance.

### GEO and citral remodels gut microbiota and suppresses meta-organismal ʟ-carnitine-TMAO metabolic pathway

**Fig. 3:**
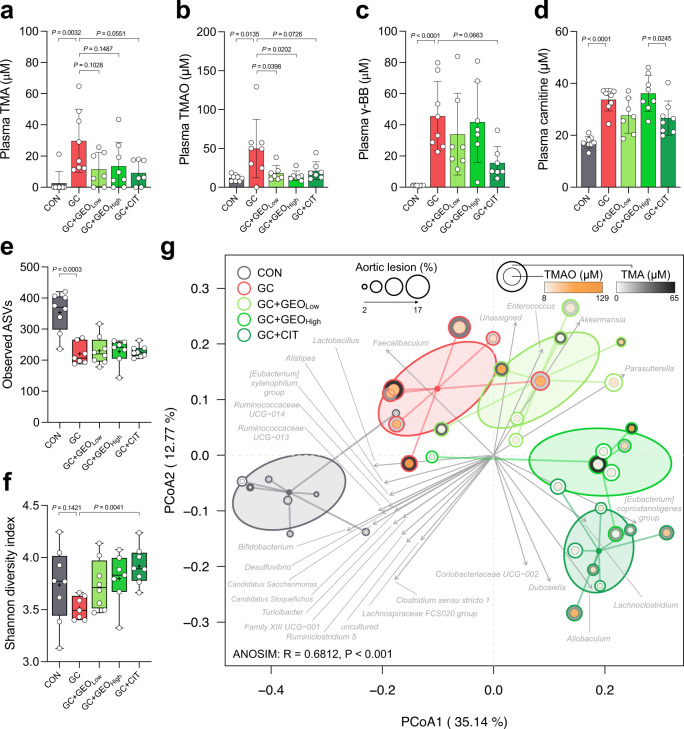
GEO and citral suppressed gut microbiota metabolites TMA and TMAO and remodeled gut microbiota composition in GC-induced atherosclerosis female ApoE^−/−^ mice. Plasma levels of (**a**) trimethylamine (TMA), (**b**) trimethylamine-N-oxide (TMAO), (**c**) γ-butyrobetaine (γ-BB), and (**d**) carnitine. **e** Observed amplicon sequencing variants (ASVs); **f** Shannon diversity index; **g** principal coordinate analysis (PCoA) plot based on Bray–Curtis dissimilarity with gut microbiota-associated vector (*n* = 7–8). Dot plots are expressed as the mean ± SD. Box plots display median, mean (+), quartiles (boxes), and range (whiskers). Statistical analyses were performed using an unpaired two-tailed Student’s *t*-test CON vs. GC groups; one-way ANOVA with Tukey’s range test for comparing GC, GC + GEO_Low_, GC + GEO_High_, and GC + CIT. Analysis of similarity (ANOSIM) was calculated to determine heterogeneity of the fecal microbiota among the groups in PCoA. Vectors in the PCoA plot indicated a significant genus (*p* < 0.001), and its length shows the strength of the correlation. CON: control diet group, GC: Gubra Amylin NASH diet [GAN diet] + ʟ-carnitine in drinking water [1.3%] group, GC + GEO_Low_: GC + Ginger essential oil (GEO) [50 mg/kg bw/day] group, GC + GEO_High_: GC + GEO [100 mg/kg bw/day] group, GC + CIT: GC + Citral [20 mg/kg bw/day] group.

TMAO is an atherosclerotic risk factor. The primary multistep meta-organismal (gut microbiota and host) pathway for TMAO production through carnitine metabolism is as follows: ʟ-carnitine → γ-butyrobetaine (γBB) → TMA → TMAO^22^. Compared with the CON group, plasma TMA (*p* = 0.0032), TMAO (*p* = 0.0135), γ-BB (*p* < 0.0001), and carnitine (*p* < 0.0001) increased by 11.2-, 4.1-, 43.3-, and 1.9-fold, respectively, in the GC group (Fig. 3a–d). However, low and high doses of GEO and citral significantly reduced plasma TMA and TMAO levels (*p* = 0.0398 and *p* = 0.0202 for low and high doses of GEO, respectively). Additionally, there was a decreasing trend in plasma γ-BB levels in the citral and GEO treatment group, with more obvious decrease observed in citral treatment group (*p* = 0.0663).

Since gut microbiota plays a critical role in the development of CVD and atherosclerosis, we examined the effect of GC, GEO, and citral on gut microbiota. To achieve this, the microbiota compositions of fecal samples were examined using V3–V4 16 S rRNA sequencing technique. The raw reads were processed using the QIIME2 pipeline to obtain the amplicon sequence variants (ASVs), which were compared against the SILVA database (version 132) for taxonomic classification. A total of 1504 ASVs were generated, which were assigned to 190 species and 122 genera. Additionally, the α-diversity, including observed amplicon sequencing variants (ASVs) and Shannon diversity index, of fecal microbiota was calculated using the vegan package in R species. Compared with the CON group, there was a decrease in observed ASVs (*p* = 0.0003) and Shannon diversity index in the GC group, indicating that GAN diet and ʟ-carnitine decreased the abundance of specific gut microbes (Fig. 3e, f). However, GEO and citral treatments increased the Shannon diversity index, with the effect of citral being significant (*p* = 0.0041).

Furthermore, the β-diversity of the gut microbiota was examined based on Bray−Curtis distance (Fig. 3g). GC, GEO, and citral affected and remodeled the fecal microbiota. Principal coordinate analysis (PCoA) showed significant separation among all groups (ANOSIM: R = 0.6812, *p* < 0.001). Specifically, the GC treatment induced a significant microbiome shift from the CON group, especially at the X-axis (PCoA1; 35.14%), indicating that the GAN/ʟ-carnitine diet altered the fecal microbiota. GEO and citral also had a secondary impact on the gut microbiome. Citral and GEO treatments induced a dose-dependent shift in microbiome from the GC group, especially at the Y-axis (PCoA2; 12.77%), with a more obvious shift in gut microbiome observed in the dot of high-dose GEO group. Furthermore, the PCoA plot provided information on the degree of aortic lesion and TMA and TMAO levels, with a larger circle indicating more severe aortic lesions and deeper colors in the circle indicating higher levels of TMA or TMAO. Consistent with the results of the histological analysis (Fig. 1c), a larger circle with deeper colors were observed in the GC group compared with the CON group; however, the GEO and citral groups tended have smaller circle sizes with lesser color intensity. Additionally, the envfit function in R package was used to elucidate the association between the genus and distance structure of the gut microbiome. Vectors in the PCoA plot represented a significant genus (*p* < 0.001), and the lengths indicated the strength of association. The CON treatment was correlated with beneficial genera, such as *Lactobacillus*, *Alistipes*, *Bifidobacterium*, and other bacteria groups, including *Eubacterium xylanophilum group*, *Ruminococcaceae UCG*−*014*, *Ruminococcaceae UCG*−*013*, *Desulfovibrio*, *Candidatus Saccharimonas*, *Candidatus Stoquefichus*, *Turicibacter*, *Family XIII UCG*−*001*, *Ruminiclostridium 5*, *Lachnospiraceae FCS020 group*, and *Clostridium sensu stricto 1*. The GC treatment was associated with *Fecalibaculum*. The low-dose GEO treatment was associated with the beneficial mucin degrading genus *Akkermansia* and other bacteria, such as *Enterococcus* and *Parasutterella*; the citral treatment was associated with CVD negatively correlated microbiomes *Allobaculum* and *Dubosiella*, and other bacteria*—*the *Coriobacteriaceae UCG*−*002*, *Lachnoclostridium*, and *Eubacterium coprostanoligenes group*. Overall, these results showed that GEO and citral potentially re-shaped the gut microbiome and decreased the meta-organismal metabolism of ʟ-carnitine by both gut microbiota and host to form TMAO.

### GEO and citral supplementation modulates gut microbiota at the genus level

**Fig. 4:**
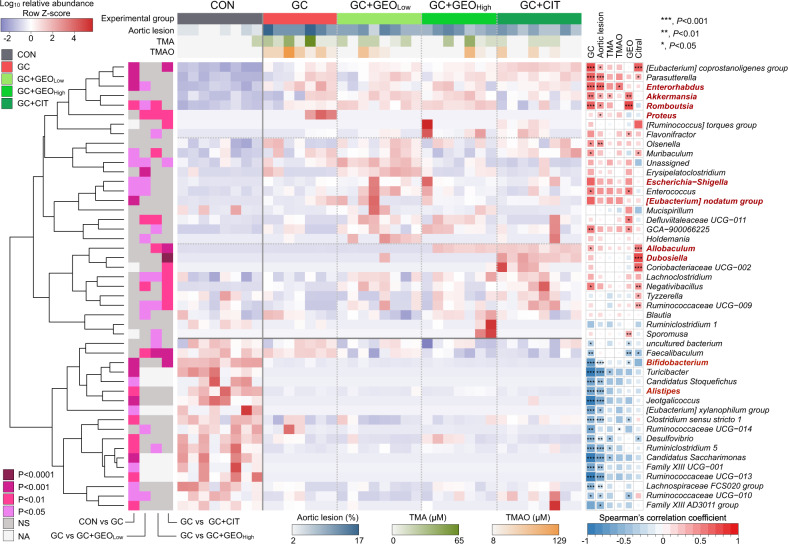
Remodeling of gut microbiota at the genus level by GC, GEO, and citral, and their relationship with aortic lesion and gut microbiota metabolites. Heatmap of the relative abundance of significantly different fecal microbiota using Kruskal–Wallis test (*p* < 0.05), and Spearman’s correlation analysis between gut microbiota components at the genus level and aortic lesion and gut microbiota metabolites. Pairwise statistical analyses were performed using an unpaired Wilcoxon signed-rank test CON vs. GC groups; Kruskal–Wallis test with Dunn’s multiple comparison test for comparing GC, GC + GEO_Low_, GC + GEO_High_, and GC + CIT. CON: control diet group, GC: Gubra Amylin NASH diet [GAN diet] + ʟ-carnitine in drinking water [1.3%] group, GC + GEO_Low_: GC + Ginger essential oil (GEO) [50 mg/kg bw/day] group, GC + GEO_High_: GC + GEO [100 mg/kg bw/day] group; GC + CIT: GC + Citral [20 mg/kg bw/day] group.

Kruskal–Wallis test was performed to identify significantly different (*p* < 0.05) genera among the five experimental groups, which were illustrated in a heatmap (Fig. 4). A total of 47 significantly different genera were identified among the treatment groups. The left panel displaying the hierarchical clustering of gut microbiota at the genus level was divided into two major clusters indicating the differences between the CON and GC groups. These data indicated that diet (control diet or GAN diet with ʟ-carnitine) was the primary factor influencing fecal microbiota at the genus level. The GC cluster consisted of 29 genera, whereas the CON cluster consisted of 18. GEO and citral treatments formed sub-clusters within the GC-induced cluster, indicating that GEO and citral played a secondary role in altering gut microbiota. The top panel indicates the degree of aortic lesions and TMA and TMAO levels in the treatment groups. Compared with the CON group, aortic lesion formation and TMA and TMAO levels were significantly higher in the GC group; however, GEO and citral treatment reversed these parameters. Statistical analyses were performed to determine significant differences between the group, and the *p* values are displayed in the left panel.

Compared with the CON group, the GC group was enriched in CVD-related bacteria, including *Enterorhabdus*, *Romboutsia*, *Proteus*, *Eubacterium nodatum group*, *Escherichia-Shigella*, *Eubacterium coprostanoligenes group*, *Parasutterella*, *Muribaculum*, and *Enterococcus*, and there was a decrease in the abundance of several beneficial microbiotas, such as *Bifidobacterium* and *Alistipes*. These data indicated that GAN diet and ʟ-carnitine negatively affected gut microbiota homeostasis by increasing CVD-associated microbiome and decreasing beneficial microbiome. Compared with the GC group, a total of 16, 13, and 13 significantly different genera were identified in the low-dose GEO, high-dose GEO, and citral groups, respectively. Interestingly, GEO treatment reduced the relative abundance of the CVD-associated bacteria *Enterorhabdus* and *Proteus* but increased the abundance of the beneficial bacteria *Allobaculum*. Additionally, citral treatment decreased the abundance of *Proteus* but increased *Allobaculum* and *Dubosiella*.

Furthermore, Spearman’s correlation analysis was performed to assess the relationship between the significant genus and CVD-related biomarkers, including aortic lesion, TMA, TMAO, GEO, and citral. Seven genera, including *Eubacterium coprostanoligenes group*, *Parasutterella*, *Enterohabdus*, *Akkermansia*, *Romboutsia, Proteus*, and *Olsenella* (a microbiome associated with CVD), were positively correlated with aortic lesions. Fifteen genera, including the beneficial bacteria *Bifidobacterium* and *Alistipes*, were negatively correlated with aortic lesions. Interestingly, CVD-related *Enterohabdus* was positively correlated with plasma TMAO levels. GEO was positively associated with a healthy microbiome consisting of *Akkermansia*, whereas citral was positively correlated with *Allobaculum* and *Dubosiella*. In summary, the heatmap and correlation data indicated that GC adversely affected gut microbiota, resulting in microbiota dysbiosis. However, GEO and citral exhibited a favorable effect and improved general gut microbiota composition, indicating that GEO and citral treatment restored gut microbiota and ameliorated atherosclerosis in GC ApoE^−/−^ mouse model.

## Discussion

In the present study, we examined the anti-atherosclerotic effect and mechanism of GEO and citral in mice. GEO and citral treatment ameliorated atherosclerosis in ApoE^−/−^ mice by suppressing meta-organismal metabolism of gut microbiota-host-derived TMA and TMAO and remodeling gut microbiota composition. Additionally, GEO and citral treatment significantly lowered plasma IL-1β, TNF-α, glucose, and insulin levels. Several studies have shown that ginger possesses cardioprotective^23^, anti-inflammatory^19^, anti-microbial^24^, and glucose-modulating functions^20^. Dietary ginger extract has been shown to suppress TMAO-induced increase in plasma cholesterol in mice and improve anti-inflammatory response by lowering plasma TNF-α, IL-1β, and IL-6 levels^18^.

Hyperlipidemia is the most crucial risk factor for atherosclerosis, which is the primary cause of CVD^25^. In the present study, GC treatment increased total cholesterol, LDL-C, and HDL-C levels, which was attributed the GAN diet (contained 2% cholesterol and 40 kcal% of fat, mainly from palm oil). The intake of palm oil-containing HFD has been shown to elevate blood cholesterol, LDL-C, and HDL-C levels^14,15^. GEO and citral treatment did not decrease total cholesterol and LDL-C levels but increased HDL-C levels, indicating their beneficial effect on blood lipid profile. Additionally, GC increased plasma glucose, insulin, and HOMA-IR levels, confirming its potential in induce metabolic syndrome, which was consistent with previous findings^14,15^. However, GEO and citral treatment significantly improved fasting blood glucose level and insulin resistance, thus improving glucose homeostasis. There was no significant difference in total plasma triglyceride levels between the groups. Since the triglyceride and cholesterol content in HFD is high, excessive cholesterol intake downregulates the production of cholesterol esters and lipoproteins in the liver, thereby inhibiting the production of triglycerides in the liver^14^. Moreover, previous studies have shown that total plasma glyceride level is not significantly affected by GAN diet^13^.

Atherosclerosis is a chronic inflammatory disease commonly manifesting as increased circulating pro-inflammatory cytokines^26^. In the present study, GC-treated ApoE^−/−^ mice had significantly higher plasma levels of pro-inflammatory cytokines, including TNF-α, IL-1β, and IL-6. IL-1β^−/−^/ApoE^−/−^ mice or TNF-α^−/−^/ApoE^−/−^ mice have been shown to have lower degree of aortic lesions, indicating the importance of IL-1β and TNF-α on aortic lesion development^27,28^. Additionally, palm oil and high cholesterol can enhance the production of IL-1β, inducing inflammation^29^. In the present study, GEO and citral treatment exhibited immunomodulatory activity by reducing plasma IL-1β and TNF-α levels.

Consuming unhealthy diet is associated with hyperlipidemia, chronic inflammation, and dysbiosis, which are major causes of CVD^3^. As earlier stated, food nutrients, such as ʟ-carnitine, can be metabolized by specific gut microbiome to form γBB and TMA^22^, and TMA can be subsequently oxidized to TMAO (the CVD risk factor) by the host hepatic flavin monooxygenase^6^. In the present study, plasma concentrations of carnitine, γ-BB, TMA, and TMAO were significantly higher in the GC group compared with the CON group. In contrast, γBB was not detected in the CON group, indicating that these metabolites are produced by the gut microbiota using ʟ-carnitine as a substrate. Apart from supplementation with ʟ-carnitine, high-fat and high-sugar diets have been shown to significantly increase plasma TMAO levels in mice^9,12^. A previous study showed that administering ʟ-carnitine alone without the HFD increased plasma TMAO level to ~20 µM^17^. Similarly, a combination of GAN diet and ʟ-carnitine increased the plasma TMAO levels of mice to approximately 50 µM in the present study. However, GEO and citral treatment significantly reduced plasma TMA and TMAO levels, indicating that they may possess antibiotic-like activity, suppressing the production of TMA-related bacteria in the gut. Moreover, GEO and citral functioned as anti-inflammatory agents to reduce the levels of circulating inflammatory markers, thus ameliorating atherosclerosis.

A previous study showed the intake of HFD altered gut microbiota in mice after 2 weeks^14^. Additionally, obesogenic and metabolic changes, NASH phenotype, altered gut microbiota composition and function, gut dysbiosis, intestinal leakage, and endotoxemia were observed in mice fed a palm oil-containing HFD^13^. In the present study, fecal microbiota ɑ- and β-diversities were altered in the GC group compared with the CON group. Specifically, there was an increase in CVD-related bacteria, including *Enterorhabdus*, *Romboutsia*, *Proteus*, *Eubacterium nodatum group*, and *Escherichia-Shigella*, and a decrease in the abundance of specific beneficial microbiomes, such as *Bifidobacterium* and *Alistipes*. Spearman’s correlation analysis showed that *Eubacterium coprostanoligenes group*, *Parasutterella*, *Enterohabdus*, *Akkermansia*, *Romboutsia*, *Proteus*, and *Olsenella* were positively correlated with aortic lesions. Additionally, *Bifidobacterium* and *Alistipes* were negatively correlated with aortic lesions, while *Enterohabdus* was positively associated with plasma TMAO levels.

ApoE^−/−^ mice fed HFD have been reported to have a higher relative abundance of *Enterorhabdus*^30^. Moreover, *Romboutsia* is more abundant in patients with normal blood pressure than in patients with hypertension^31^, making *Romboutsia* an indicator of systolic blood pressure^32^. *Proteus penneri* and *Escherichia fergusonii* are well-known CVD-related bacteria that can convert ʟ-carnitine to γBB^33^. Particularly, the *Eubacterium nodatum group* and *Emergencia timonensis* can anaerobically metabolize γ-BB to TMA^22^. In contrast, probiotics, such as *Bifidobacterium breve* and *Bifidobacterium longum*, improves cardiovascular health by modulating gut microbiota and reducing plasma TMAO level in choline-fed mice^34^. *Alistipes*, short-chain fatty acid (SCFA)-producing bacteria genera, were significantly enriched in healthy volunteers than in NASH patients, indicating that the bacteria can play a beneficial role against liver diseases by secreting SCFA. However, *Alistipes* are associated with hypertension^35^. A combination of GAN diet and ʟ-carnitine may negatively affect gut microbiota composition, resulting in dysbiosis.

In the present study, GEO and citral treatment affected the ɑ- and β-diversities of gut microbiota. Interestingly, GEO treatment decreased the abundance of the CVD-associated bacteria, including *Enterorhabdus* and *Proteus*, but increased the abundance of the beneficial bacteria *Allobaculum*. Additionally, citral treatment also decreased the abundance of *Proteus* and enriched *Allobaculum* and *Dubosiella*. Moreover, GEO was positively correlated with *Akkermansia*, whereas citral was positively associated with *Allobaculum* and *Dubosiella*.

*Akkermansia muciniphila* prevents atherosclerosis in ApoE^−/−^ mice by decreasing endotoxemia and inflammation^36^. Additionally, ginger extract increased the relative abundance of *Allobaculum* in mice fed HFD; moreover, transplantation with fecal contents from mice fed HFD supplemented with ginger extract increased the abundance of *Allobaculum*^37^. Furthermore, the abundance of *Allobaculum* is lower in ApoE^−/−^ mice fed HFD, indicating a negative correlation between *Allobaculum* and atherosclerosis^38^. Hypertensive mice had a low abundance of *Dubosiella*, which was negatively correlated with CVD^39^. Previous studies have shown that GEO remodeled gut microbiota and reversed dysbiosis to amelioration NASH in a murine model of NASH; moreover, GEO suppressed the NLRP3 inflammasome and mediated the gut microbiota-LPS-TLR4 pathway^20^. Collectively, GEO and citral treatments improved gut microbiota composition in the ApoE^−/−^ mice. Several foods and herbs have been shown to prevent CVD through gut microbiota modulation^3^. Berberine, a natural alkaloid found in *Coptis chinensis* and *Berberis vulgaris*, has been reported modify gut microbiota composition and functionality, inhibit TMA and TMAO production, and alleviate atherosclerosis^38^. Another study found that the intake of raw garlic juice for 2 weeks reduced gut microbiota-host-derived TMAO and increased the abundance of specific beneficial bacteria in humans. Moreover, the active component of raw garlic, known as allicin, has been showed to reduce circulating TMA and TMAO levels and ameliorating atherosclerosis in ApoE^−/−^ mice fed carnitine via gut microbiota modulation^17^.

**Fig. 5:**
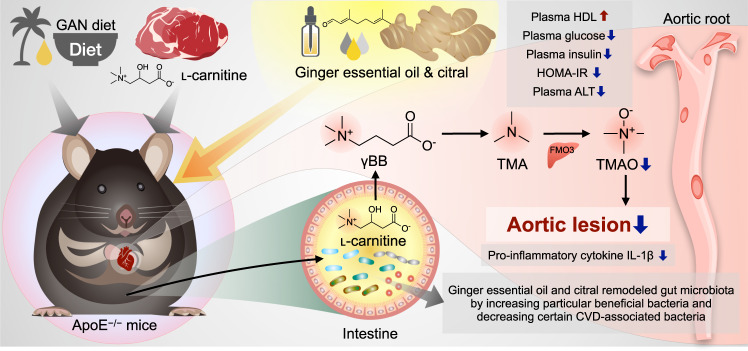
GEO and citral prevents cardiovascular disease and ameliorates atherosclerosis through gut microbiota and TMAO modulation. GEO and citral (1) protected ApoE^−/−^ mice from GC-induced atherosclerosis and improved plasma lipidemic biomarker HDL-C; (2) enhanced glucose and insulin homeostasis, ameliorated hepatic damage, and reduced plasma levels of pro-inflammatory cytokine IL-1β; (3) remodeled gut microbiota and downregulated meta-organismal metabolism of the ʟ-carnitine-TMAO pathway; and (4) favorably modulated the gut microbiota. GC: Gubra Amylin NASH diet [GAN diet] with ʟ-carnitine in drinking water, GEO: ginger essential oil, TMAO: trimethylamine-N-oxide.

In summary, the results of this study showed that GEO and citral exhibit cardio-protective effects by modulating gut microbiota, inhibiting the formation of TMAO, reducing pro-inflammatory cytokine levels, and improving insulin resistance (Fig. 5), indicating that GEO and citral may serve as potential dietary supplements for CVD prevention.

## Methods

### GEO extraction and analysis of its major components

Ginger (*Zingiber officinale* Roscoe) samples were purchased from Nantou Mingjian Country Farmers Association, Nantou, Taiwan, washed, cut into slices, and blended with three volumes of distilled water. GEO was then extracted from the resulting puree for approximately 6 h using steam distillation, yielding a yellowish clear essential oil. The concentration of the extract was approximately 0.14% (w/w), and the chemical constituent was analyzed using Thermo Scientific Focus gas chromatography equipped with an AI 3000 II autosampler and a flame ionization detector. The analysis program and conditions are as follows: flow rate of the carrier N_2_ gas was 1 mL/min with a split ratio of 40:1; initial column temperature, 50 °C; final column temperature, 200 °C; an increase of 7 °C/min to 200 °C held for 0 min; injector temperature, 250 °C; detector temperature, 250 °C; and injection volume, 0.3 μL. Citral (purity > 95%, Sigma-Aldrich, USA) was used as a standard. A representative chromatogram is shown in Supplementary Fig. 1. The primary component of GEO was citral, comprised of a mixture of the two geometric isomers, geranial and neral. The retention times of geranial and neral were 17.04 min and 17.82 min, respectively, and the area under curve (AUC) was calculated to quantify the amount of both components in GEO. Citral accounted for approximately 31% (neral: 18.8% and geranial: 12.2%) of constituents of GEO, which was consistent with previous findings (30%)^19^.

### Animal model

ApoE^−/−^ mice were originally purchased from the Jackson Laboratory (Bar Harbor, ME) and bred in the animal house of Institute of Food Science and Technology, National Taiwan University. The animals were handled according to the guidelines established by the Institutional Animal Care and Use Committee of National Taiwan University (Approval No: NTU-109-EL-00131). Eight-week-old C57BL/6 ApoE^−/−^ female mice were housed in a room at 22 ± 2 °C under a 12 h light/dark cycle. Compared with male ApoE^−/−^ mice, female ApoE^−/−^ mice exhibited pronounced flavin monooxygenase 3 (FMO3) activity and significantly elevated plasma TMAO levels; hence, the female mice were selected for this study^40^. After adaptation for 2 weeks, the mice were randomly divided into five groups with eight mice in each: (1) CON (control diet); (2) GC (GAN diet + 1.3% ʟ-carnitine in drinking water); (3) GC + GEO_Low_ (50 mg/kg bw/day); (4) GC + GEO_High_ (100 mg/kg bw/day); (5) GC + CIT [GC + Citral (20 mg/kg bw/day)]. The control diet contained 10 kcal% fat (Research Diets, Inc., NJ, USA; D12450K), while the GAN diet contained 40 kcal% fat, 20 kcal% fructose, and 2% cholesterol (GAN diet; Research Diets, Inc., NJ, USA; D09100310)^14^. GEO and citral were dissolved in soybean oil. Mice in the CON and GC groups were also given soybean oil via oral gavage method, and the dosage of GEO and citral and the composition of citral as neral in GEO used in this study was based on previous studies^19^. All mice were fed the experimental diets and liquid ad libitum. After 16 weeks, the mice were sacrificed using carbon dioxide asphyxiation and blood was collected by cardiac puncture using a syringe.

### Oil red O staining of the aorta

Aorta samples collected from the mice were rinsed with phosphate-buffered saline to clean residual blood and dissected under the microscope using a micro-scissor and tweezers to remove the fatty tissues. The dissected sample was fixed in 10% formalin overnight, washed with distilled water for 5 min, soaked twice in propylene glycol for 5 min, stained with oil red O for approximately 10 min, soaked in 85% propylene glycol for 3 min, and washed again with distilled water for 3 min. Images of the stained aorta were captured under a microscope and aortic lesions were quantified using Image J software (Version 1.8.0)^17^.

### Plasma biochemical analysis

Blood samples from the mice were centrifuged at 1000 × *g* for 15 min (4 °C) to extract plasma. The total cholesterol, total triglyceride, HDL-C, glucose, AST, and ALT contents of the plasma samples were determined using commercial test strips in an automatic blood analyzer (SpotchemTM II reagent strip; Arkray Inc., Kyoto, Japan)^17^. LDL-C was calculated using the Friedewald equation^41,42^. ox-LDL was measured using a commercial enzyme-linked immunosorbent assay (ELISA) kit (CSB-E07933m, Cusabio Biotech Co., Ltd., China)

### Analysis of plasma concentrations of TMA, TMAO, γBB, and carnitine using liquid chromatography-mass spectrometry

Briefly, 5 μL of plasma was mixed with 20 μL of deionized water, and 10 μL of this mixture was then added to 190 μL of isotopically labeled internal standards (d_3_-carnitine^13^,C_3_-TMA, and ^13^C_3_-TMAO) in 0.1% formic acid acetonitrile solution. The solution was subsequently centrifuged at 12,000 × *g* for 5 min at 4 °C to separate the supernatant. Subsequently, 100 μL of the supernatant was used for the quantification of TMA, TMAO, γBB, and carnitine levels using liquid chromatography-tandem mass spectrometry (LC-MS/MS; EXION LC, ABSCIEX with TripleQuad 5500, ABSCIEX).

For LC-MS/MS analysis, 5 μL of each plasma sample was injected into an ABSCIEX EXION LC system coupled with an ABSCIEX TripleQuad 5500 mass spectrometer (AB SCIEX, Canada). The separation was performed using an ACQUITY UPLC BEH Amide column (2.1 × 150 mm, 1.7 μm, Waters, USA) maintained at 40 °C. Mobile phase A was 0.1% formic acid in deionized water, and mobile phase B was 0.1% formic acid in acetonitrile; flow rate was 0.15 mL/min. The LC program was as follows: 0–1 min, 50% solvent B; 1–2 min, 50–40% solvent B; 2–3 min, 40–50% solvent B; and 3–5 min, 50% solvent B. The electrospray was set in positive ionization mode with the following parameters: curtain gas supply, 50 psi; capillary temperature, 500 °C; spray voltage rating, 5 kV. The detected peak area ratio was used to calculate the concentration of each target analyte in the plasma sample against the calibration curve^17^.

### Assessment of insulin resistance

Plasma insulin levels were measured using a commercial ELISA kit (Mercodia mouse insulin ELISA kit), according to the manufacturer’s instructions (10-1247-01, Mercodia Inc, USA). Additionally, Homeostatic Model Assessment-Insulin Resistance index (HOMA-IR) was calculated to determine the effect of the treatments on insulin resistance using the following formula:

HOMA-IR = [fasting insulin (mU/L) × fasting glucose (mmol/L)]/22.5^43^.

### Analysis of plasma levels of pro-inflammatory cytokines

The plasma levels of pro-inflammatory cytokines, including TNF-α, IL-1β, and IL-6, were measured using a commercial ELISA kit (Invitrogen, USA), according to the manufacturer’s instructions^19^.

### Gut microbiota composition analysis

Mouse fecal contents were collected from the large intestine, snap frozen in liquid nitrogen, and stored at −80 °C before use. Fecal samples were used for DNA extraction, V3-V4 region 16S rRNA gene amplification, and construction of the sequencing library. Fecal genomic DNA was extracted using QIAamp Power Fecal Pro DNA Kit (QIAGEN, Netherlands), according to the manufacturer’s instructions. The V3-V4 region of the 16S rRNA gene was amplified on a polymerase chain reaction system using forward and reverse primer pairs [(Forward = 5′-TCG TCG GCA GCG TCA GAT GTG TAT AAG AGA CAG CCT ACG GGN GGC WGC AG-3′) and (Reverse = 5′-GTC TCG TGG GCT CGG AGA TGT GTA TAA GAG ACA GGA CTA CHV GGG TAT CTA ATC C-3′)]. The PCR conditions were as follows: initial denaturation at 95 °C for 3 min, followed by 25 cycles at 95 °C for 30 s, 55 °C for 30 s, 72 °C for 30 s, and a final extension at 72 °C for 5 min. The final amplified products were subsequently visualized using 2% agarose gel electrophoresis. Library sequencing was performed on the Illumina MiSeq platform, according to manufacturer’s instructions. The raw sequences were processed according to the QIIME2 pipeline, and the amplicon sequence variant (ASV) table was prepared according to the SILVA database (version 132). α-diversity, including observed species and Shannon diversity index, was calculated using the vegan package in R software. Principal coordinates analysis (PCoA) was performed based on Bray-Curtis distance. Analysis of similarity (ANOSIM) was performed to determine the heterogeneity of the fecal microbiota among all groups. Heatmap and correlation were plotted using the heatmap3 and corrplot packages in R, respectively^17^.

### Statistical analyses

All data were presented as mean ± standard deviation (SD). An unpaired two-tailed Student’s t-test or one-way analysis of variance (ANOVA) with Tukey’s range test was used to compare the group means. Wilcoxon signed-rank test, Kruskal–Wallis test, Dunn’s multiple comparison test, unpaired two-tailed Student’s t-test, and one-way ANOVA with Tukey’s range test were used to analyze the fecal microbiome data based on whether the datasets were normally distributed. All statistical analyses were performed using the Graphpad Prism (version 9.4.1) or R (version 3.6.1).

### Reporting summary

Further information on research design is available in the Nature Research Reporting Summary linked to this article.

## Supplementary information

Supplementary Information

Reporting Summary

## Data Availability

The raw 16S rRNA sequencing data used to produce all figures are accessible at the NCBI Short Read Archive under the following accession numbers: BioProject: PRJNA894809, BioSample: SAMN31475470, and SRA: SRR22062157-78.

## Code availability

Bioinformatic tools, software version, parameters, and open-source code used in this present study are described in the “Methods” section. More details regarding the code to reproduce the analyses are available upon request.
