# Supplementary material for: Ginger essential oil and citral ameliorates atherosclerosis in ApoE−/− mice by modulating trimethylamine-N-oxide and gut microbiota
Source: NPJ Sci Food. 2023 May 20;7:19. doi: 10.1038/s41538-023-00196-0 (PMC10199921; doi:10.1038/s41538-023-00196-0)
Supplement: Supplementary file 1 — Supplementary Information [file 41538_2023_196_MOESM1_ESM.pdf]

Supplementary Information

Ginger essential oil and citral ameliorate atherosclerosis via modulating TMAO and gut microbiota in ApoE$^{-/-}$ mice fed on Gubra amylin NASH diet with L-carnitine

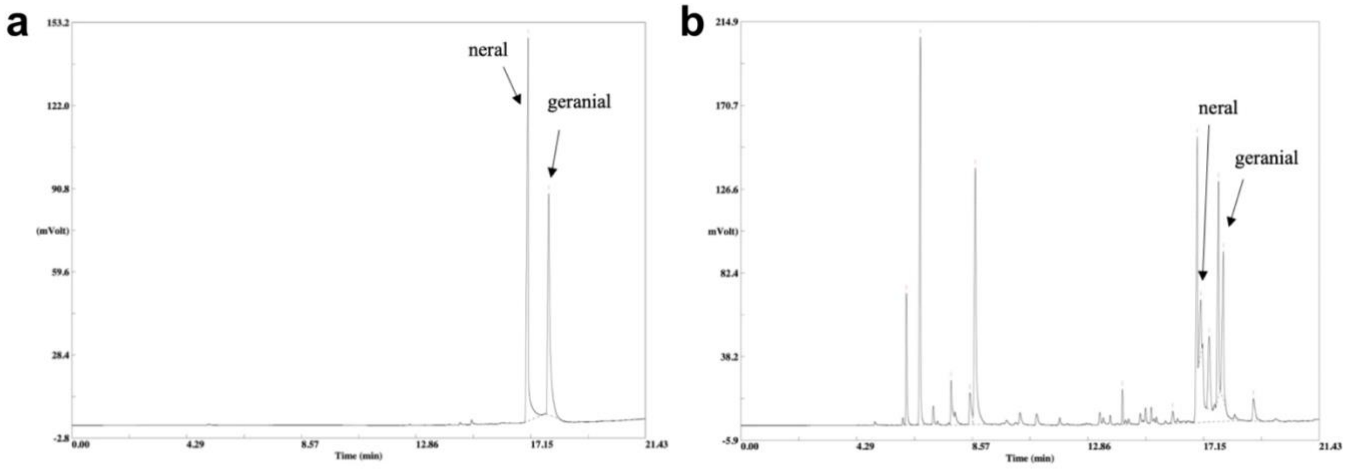

Supplementary Fig 1| Gas chromatographic data identified citral as the major compound in ginger essential oil (GEO). (a) Citral standard comprised of a mixture of the two geometric isomers geranial and neral, and (b) ginger essential oil (GEO) isolated from *Zingiber officinale* by steam distillation.
