# Supplementary material for: Ginger essential oil and citral ameliorates atherosclerosis in ApoE−/− mice by modulating trimethylamine-N-oxide and gut microbiota
Source: NPJ Sci Food. 2023 May 20;7:19. doi: 10.1038/s41538-023-00196-0 (PMC10199921; doi:10.1038/s41538-023-00196-0)
Supplement: Supplementary file 2 — Reporting Summary [file 41538_2023_196_MOESM2_ESM.pdf]

nature portfolio

Corresponding author(s): Lee-Yan Sheen, Ming-Shiang Wu

Last updated by author(s): Jan 15, 2023

Reporting Summary

Nature Portfolio wishes to improve the reproducibility of the work that we publish. This form provides structure for consistency and transparency in reporting. For further information on Nature Portfolio policies, see our Editorial Policies and the Editorial Policy Checklist.

Statistics

For all statistical analyses, confirm that the following items are present in the figure legend, table legend, main text, or Methods section.

n/a	Confirmed	
☐	☒	The exact sample size (*n*) for each experimental group/condition, given as a discrete number and unit of measurement
☐	☒	A statement on whether measurements were taken from distinct samples or whether the same sample was measured repeatedly
☐	☒	The statistical test(s) used AND whether they are one- or two-sided<br>*Only common tests should be described solely by name; describe more complex techniques in the Methods section.*
☒	☐	A description of all covariates tested
☐	☒	A description of any assumptions or corrections, such as tests of normality and adjustment for multiple comparisons
☐	☒	A full description of the statistical parameters including central tendency (e.g. means) or other basic estimates (e.g. regression coefficient) AND variation (e.g. standard deviation) or associated estimates of uncertainty (e.g. confidence intervals)
☐	☒	For null hypothesis testing, the test statistic (e.g. *F*, *t*, *r*) with confidence intervals, effect sizes, degrees of freedom and *P* value noted<br>*Give P values as exact values whenever suitable.*
☒	☐	For Bayesian analysis, information on the choice of priors and Markov chain Monte Carlo settings
☒	☐	For hierarchical and complex designs, identification of the appropriate level for tests and full reporting of outcomes
☐	☒	Estimates of effect sizes (e.g. Cohen's *d*, Pearson's *r*), indicating how they were calculated

Our web collection on statistics for biologists contains articles on many of the points above.

Software and code

Policy information about availability of computer code

Data collection	No software was used for data collection.
Data analysis	All statistics were analyzed using Graphpad Prism (version 9.4.1), R (version 3.6.1), or R Studio (version 1.2.5001). The 16S rRNA raw sequences were processed by following QIIME2 pipeline as described a detailed in the method section.

For manuscripts utilizing custom algorithms or software that are central to the research but not yet described in published literature, software must be made available to editors and reviewers. We strongly encourage code deposition in a community repository (e.g. GitHub). See the Nature Portfolio guidelines for submitting code & software for further information.

Data

Policy information about availability of data

All manuscripts must include a data availability statement. This statement should provide the following information, where applicable:

- Accession codes, unique identifiers, or web links for publicly available datasets
- A description of any restrictions on data availability
- For clinical datasets or third party data, please ensure that the statement adheres to our policy

The raw 16S rRNA sequencing data used to produce all figures are accessible at the NCBI Short Read Archive under the following accession numbers: BioProject: PRJNA894809, BioSample: SAMN31475470, and SRA: SRR22062157-78.

Field-specific reporting

Please select the one below that is the best fit for your research. If you are not sure, read the appropriate sections before making your selection.

☒ Life sciences ☐ Behavioural & social sciences ☐ Ecological, evolutionary & environmental sciences

For a reference copy of the document with all sections, see nature.com/documents/nr-reporting-summary-flat.pdf

Life sciences study design

All studies must disclose on these points even when the disclosure is negative.

Sample size	No statistical methods were used to predetermine the sample size in this study.
Data exclusions	No data were excluded from the analysis.
Replication	Experimental replications were addressed in the figure legend.
Randomization	The mice were randomly divided into their experimental group before the intervention began.
Blinding	There was no blinding necessary.

Reporting for specific materials, systems and methods

We require information from authors about some types of materials, experimental systems and methods used in many studies. Here, indicate whether each material, system or method listed is relevant to your study. If you are not sure if a list item applies to your research, read the appropriate section before selecting a response.

Materials & experimental systems

n/a	Involved in the study	
☒	☐	Antibodies
☒	☐	Eukaryotic cell lines
☒	☐	Palaeontology and archaeology
☐	☒	Animals and other organisms
☒	☐	Human research participants
☒	☐	Clinical data
☒	☐	Dual use research of concern

Methods

n/a	Involved in the study	
☒	☐	ChIP-seq
☒	☐	Flow cytometry
☒	☐	MRI-based neuroimaging

Animals and other organisms

Policy information about studies involving animals; ARRIVE guidelines recommended for reporting animal research

Laboratory animals	Eight-week-old C57BL/6 ApoE−/− female mice
Wild animals	No wild animal was involved in this study.
Field-collected samples	This study did not involve field-collected samples.
Ethics oversight	The handling of animals complied with the guidelines of the Institutional Animal Care and Use Committee of the National Taiwan University (approval number: NTU-109-EL-00131).

Note that full information on the approval of the study protocol must also be provided in the manuscript.
